# TCM and related active compounds in the treatment of gout: the regulation of signaling pathway and urate transporter

**DOI:** 10.3389/fphar.2023.1275974

**Published:** 2023-11-29

**Authors:** Xin Sun, Le Yang, Hui Sun, Ye Sun, Shuyun Wei, Ying Han, Wenkai Wang, Ling Kong, Xijun Wang

**Affiliations:** ^1^ State Key Laboratory of Dampness Syndrome of Chinese Medicine, The Second Affiliated Hospital of Guangzhou University of Chinese Medicine, Guangzhou, China; ^2^ State Key Laboratory of Integration and Innovation of Classical Formula and Modern Chinese Medicines, National Chinmedomics Research Center, Heilongjiang University of Chinese Medicine, Harbin, China

**Keywords:** gout, TCM, signaling pathway, urate transporter, review

## Abstract

Gout represents a metabolic ailment resulting from the accumulation of monosodium urate crystals within joints, causing both inflammation and, harm to tissues. The primary contributor to gout’s emergence is an elevated presence of serum urate, which is under the regulation of kidney and, gut urate transporters. Mitigating this risk factor is crucial for averting gout’s onset. Several treatments rooted in TCM and related active compounds have demonstrated efficacy in managing gout, skillfully regulating serum uric acid (UA) levels and curbing inflammation’s progression. This analysis compiles key foundational research concerning the molecular signaling pathways and UA transporters linked to gout, under the regulation of TCM. The focus includes individual botanical drug, active compounds, and TCM formulations, which have been consolidated and examined in this overview. The primary keywords chosen were “gout, hyperuricemia, gouty arthritis, traditional Chinese medicine, Chinese botanical drug, medicinal botanical drug, and natural plant”. Various relevant literature published within the last 5 years were gathered from electronic databases, including PubMed, Web of Science, CNKI, and others. The findings revealed that TCM has the capacity to modulate various signaling pathways, including MAPK, NF-κB, PI3K/Akt, NLRP3 and JAK/STAT. Additionally, it impacts UA transporters like URAT1, GLUT9, ABCG2, as well as OATs and OCTs, thereby contributing to gout treatment. TCM helps maintain a balanced inflammatory interaction and facilitates UA excretion. This study enhances our understanding of TCM’s anti-gout mechanisms and introduces novel perspectives for establishing the clinical significance and future prospects of TCM-based gout treatment.

## Graphical Abstract

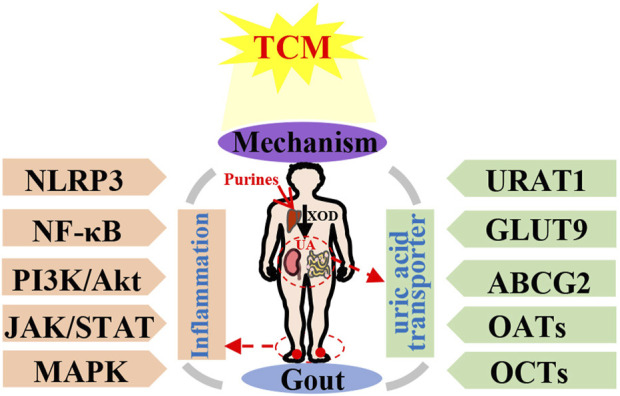

## 1 Introduction

Gout represents a painful type of arthritis triggered by the accumulation of monosodium urate (MSU) crystals within synovial fluid and other bodily tissues in people with hyperuricaemia. Intermittent flaring condition of gout often bring along unforeseeable discomfort, swelling of joints, reddening, and damage to joints as a result of recurrent flare-ups (Richette et al., 2020). The enhancement of people’s material living conditions and the escalating consumption of purine-rich foods have driven a year-by-year surge in the prevalence of gout. At present, many the global populace grapples with gout, transforming it into a significant quandary for public health, inflicting immense anguish and imposing substantial financial burdens on sufferers (Shields and Beard, 2015). Currently, the acute manifestation of gout has been addressed through the utilization of corticosteroids, colchicines, and non-steroidal anti-inflammatory medications (Qaseem et al., 2017). However, these accessible treatment options merely mitigate symptoms or delay the onset of gout, and even entail significant adverse effects, such as gastrointestinal responses, skin eruptions, systemic gaps, and the potential for renal dysfunction (Perez-Ruiz et al., 2015). Therefore, the extended management of gout necessitates the swift development of novel and more dependable therapeutic strategies.

**FIGURE 1:**
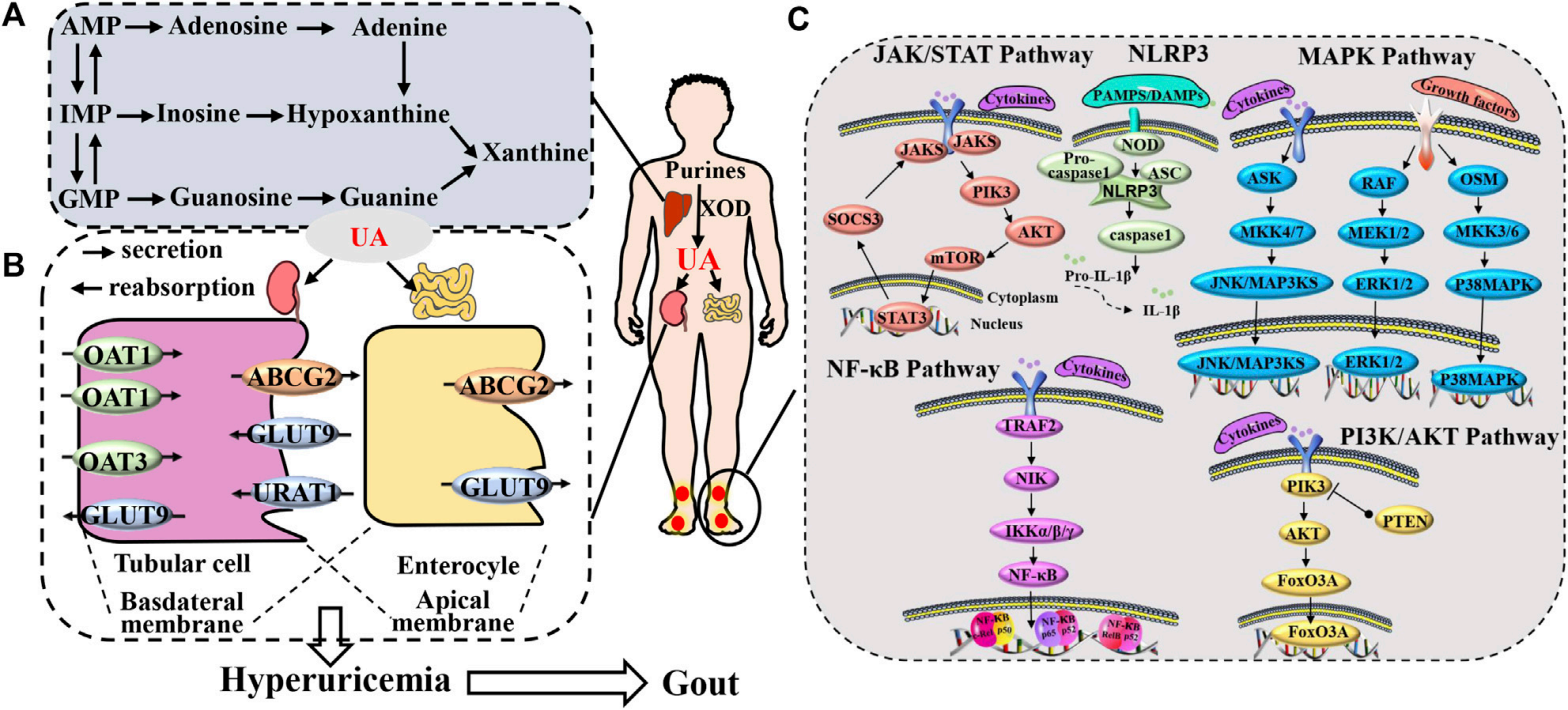
Gout pathogenesis. **(A)** The production process of uric acid. **(B)** process of uric acid excretion mediated by uric acid transporters and **(C)** the gout inflammation.

Gout presents a multifaceted pathological sequence, encompassing diverse underlying factors. These include urate accumulation (Maiuolo et al., 2016), hindrances to uric acid elimination (Méndez-Salazar and Martínez-Nava, 2022), and inflammatory response triggered by deposited urate (Shin et al., 2019), as well as the interactions among these factors (Merriman, 2015; Major et al., 2018). UA forms as a byproduct of human purine metabolism. Purines undergo a metabolic process leading from hypoxanthine to xanthine through the action of xanthine dehydrogenase, and finally to UA. Furthermore, excessive purine intake arises from the consumption of items rich in purine, including foods abundant in both purine and high sugar or alcohol content. These dietary choices prompt the assimilation of substantial adenosine triphosphate (ATP), resulting in the generation of adenosine diphosphate (ADP) and adenosine monophosphate (AMP). This, in turn, elevates AMP deaminase activity, facilitating the conversion of AMP into inosine monophosphate (IMP), thus expediting UA production (Li et al., 2020c; Zhang et al., 2020). On a different note, the excretion of UA is under the regulation of both the renal and intestinal systems. Inadequate UA elimination gives rise to an elevation in serum UA levels (Li et al., 2023b). The kidney is responsible for excreting UA through a process involving glomerular filtration, proximal tubule reabsorption, as well as secretion and subsequent reabsorption (García-Arroyo et al., 2018). The proper uptake and release of UA rely on transport proteins located in the apical and basolateral membranes of the epithelial cells in the proximal tubules. These proteins could potentially give rise to irregularities, culminating in anomalous UA elimination and consequently elevating the concentration of UA in the bloodstream (Joosten et al., 2020). Moreover, UA holds the capacity to trigger numerous intracellular signaling pathways, culminating in the generation of inflammatory cytokines (Zhang et al., 2021a; Cobo et al., 2022; Zaninelli et al., 2022). This, in turn, culminates in an inflammatory response and tissue deterioration, where inflammatory cells and factors synergistically incite each other, contributing to the progression of gout (Zhang et al., 2021c; Zhu et al., 2021). Collectively, these consequences of urate are believed to underlie a significant portion of the physiological implications of hyperuricemia and gout, highlighting that this biologically active molecule surpasses its mere status as a ‘byproduct’ of purine metabolism (Figure 1).

TCM boasts a legacy of millennia in China (Zhang et al., 2018a). A wealth of clinical and laboratory research endeavors have been undertaken to validate the efficacy of TCM in addressing gout (Xiao et al., 2018; Liu et al., 2021; Xiao et al., 2021). Certain Chinese medicinal botanical drugs or TCM formulations demonstrate multifaceted targeting and low toxicity. As a result, they present favorable characteristics and hold promising potential for both preventing and treating gout **(** Table 1). For instance, extracts like *Phellodendron chinense* C.K.Schneid. (Lin et al., 2021), *Coptis chinensis* Franch. (Li et al., 2021a), *Atractylodes lancea* (Thunb.) DC. (Fu et al., 2021), *Achyranthes bidentata* Blume. (Wu et al., 2018), *Smilax glabra* Roxb. (Liang et al., 2019), *Eucommia ulmoides* Oliv. (Fang et al., 2019), Ermiao Wan (Gu et al., 2023), Simiao Wan (Lin et al., 2020a), Sanmiao Wan (Wu et al., 2018), Wuling San (Huang et al., 2023) and Erding Formula (Zuo et al., 2018) demonstrated efficacy in enhancing gout-induced pathological damage and reducing inflammatory responses.

**TABLE 1:** Anti-gout active ingredients of Chinese botanical drug.

Classifications	Components	Chinese botanical drug	Refs
flavonoids	Scutellarin	Dry whole herbs of *Erigeron breviscapus* (Vaniot) Hand.-Mazz [Lamiaceae; Scutellariae radix]	Li et al. (2020b)
	Naringenin	Dry immature fruits of *Citrus aurantium* L. or Dry immature fruits of *Citrus sinensis* Osbeck [Rutaceae; aurantii fructus immaturus]	Yang et al. (2023)
	Genistein	Dry rhizomes of *Genista tinctoria* L	Wei-Yun and Cailin (2021)
	Apigenin	Dry ripe seeds of *Plantago asiatica* L. [Plantasinaceac; Plantaginis semen]	Liu et al. (2022a)
	The ethanol extract of Smilacis glabrae rhizoma	Dry rhizomes of *Smilax glabra* Roxb. [Liliaceae; Smilacis glabrae rhizoma]	Huang et al. (2019)
	Luteolin	Dry whole *Lagotis Brachystachya* Maxim	Zhu et al. (2021)
	α-Viniferin	Flowers of *Caragana sinica* (Buc’hoz) Rehd	Guo et al. (2023)
	The ethanol extract of *Moringa Oleifera* Lam	Leaves of *Moringa Oleifera* Lam	Luo et al. (2022)
	Epigallocatechin gallate	Leaves of *Camellia sinensis* (L.) Kuntze	Li et al. (2020a)
	Hesperetin	Dry immature fruits of *Citrus aurantium* L. [Rutaceae; urantii fructus] or dry immature fruits of *Citrus sinensis* Osbeck [Rutaceae; aurantii fructus immaturus]	An et al. (2023)
	Anthocyanins	Tuber of *Bletilla striata* (Thunb.) Rchb.f. [Orchidaceae; Bletillae rhizome]	Qian et al. (2019)
	Mangiferin	leaves, fruits and barks of *Mangifera indica* L and leaves *Anemarrhena asphodeloides* Bunge [Liliaceae; Anemarrhenae rhizoma]	Li et al. (2020d)
	Chrysin	The extract of honey, propolis, and mushrooms	Chang et al. (2021)
	Baicalein	Dry roots of *Scutellaria baicalensis* Georgi [Liliaceae; Smilacis glabrae rhizoma]	Chen et al. (2021)
	Phloretin	The extract of the *Prunus mandshurica* (Maxim.) Koehne [Rosaceae; Armeniacae semen amarum]	Cui et al. (2020)
Alkaloids	Tetrahydropalmatine	Dry tubers of *Corydalis yanhusuo* W.T.Wang [Papaveraceae; Corydalis rhizoma]	Wang et al. (2021e)
	Palmatine	Dry barks of *Phellodendron chinense* C.K.Schneid. [Rutaceae; Phellodendri chinensis cortex]	Cheng et al. (2022)
	Berberine	Dry barks of *Phellodendron amurense* Rupr.Guan [Rutaceae; Phellodendri amurensis cortex]	Li et al. (2021a)
	Dihydroberberine	Dry barks of *Phellodendron chinense* C.K.Schneid. [Rutaceae; Phellodendri chinensis cortex]	Xu et al. (2021)
	Coptisine	Dry rhizomes of *Coptis chinensis* Franch. [Ranunculaceae; Coptidis rhizoma]	Liu et al. (2022b)
	Nuciferine	Leaves of *Nelumbo nucifera* Gaertn	Zhang et al. (2022b)
Quinones	Tanshinone IIA	Dry roots and rhizomes of *Salvia miltiorrhiza* Bunge. [Lamiaceae; Salviae miltiorrhizae radix et rhizoma]	Xu et al. (2022b)
	Rhein	Dry roots and rhizomes of *Rheum palmatum* L [Polygonaceae; Rhei radix et rhizoma]	Chang et al. (2019)
Polyphenols	Curcumin	Dry rhizomes of *Curcuma Longa* L. [Zingiberaceae; Curcumae longae rhizoma]	Chen et al. (2019d)
	Paeonol	Dry roots of *Paeonia lactiflora* Pall. [Ranunculaceae; Paeoniae radix alba] or dry barks of *Paeonia × suffruticosa* Andrews [Ranunculaceae; Moutan cortex]	Chen et al. (2019c)
	Resveratrol	Dry roots and rhizomes of *Polygonum cuspidatum* Siebold & Zucc [Polygonaceae; Polygoni cuspidati rhizoma et radix] or dry roots of *Veratrum grandiflorum* (Maxim. ex Miq.) O.Loes	Li et al. (2019b)
	Bergenin	Dry grass of *Bergenia crassifolia* (L.) Fritsch	Chen et al. (2020)
	Chlorogenic acid	Aabastrums of *Lonicera japonica* Thunb. [Caprifoliaceae; Lonicerae japonicae flos]	Zhou et al. (2021)
Volatile oils	β-caryophyllene	Dry ripes Alabastrum of *Eugenia caryophyllata* Thunb. [Myrtaceae; Caryophylli flos]	Li et al. (2021b)
	Carvactol	Dry overground parts of *Mosla chinensis* Maxim. [Lamiaceae; Moslae herba]	Riaz et al. (2022)
Phenylpropanoids	Esculetin	Branch barks of *Fraxinus rhynchophylla* Hance [Oleaceae; Fraxini cortex]	Wang et al. (2020c)
	Chicoric acid	*Cichorium intybus* L and the echinacea plant	Wang et al. (2021b)
Glucosides	Icariin	Dry leaves of *Epimedium brevicornu* Maxim. [Berberidaceae; Epimedii folium]	Cao (2021)
	Geniposide	Dry ripe fruits of *Gardenia jasminoides* J.Ellis [Rubiaceae; Gardeniae fructus]	Wang et al. (2022a)
	The extract of *Paeoniae radix* alba	Dry roots of *Paeonia lactiflora* Pall. [Ranunculaceae; Paeoniae radix alba]	Meng et al. (2021)
	Dioscin	Dry rhizomes of *Dioscorea hypoglauca* Palib [Dioscoreaceae; Dioscoreae hypoglaucae rhizoma]	Zhou et al. (2018)
	Eurycomanol	Dry stems of *Eurycoma longifolia* Jack male fertility	Bao et al. (2022)
Saponins	The ethanol extract of *Chenopodium quinoa* Willd	Dry ripe fruits of *Chenopodium quinoa* Willd	Lin et al. (2023)
	The ethanol extract of *Dioscorea polystachya* Turcz	Dry rhizomes of *Dioscorea polystachya* Turcz	Zhou et al. (2020)
	Celastrol	Dry roots of *Tripterygium wilfordii* Hook. f	Yan et al. (2021)
Others	Polysaccharide	Dry roots of *Isatis indigotica* Fortune ex Lindl. [Brassicaceae; Isatidis radix]	Ren et al. (2022)
	The ethanol extract of Plantaginis semen	Dry ripe seeds of *Plantago asiatica* L. [Plantasinaceac; Plantaginis semen]	Yang et al. (2021a)
	The ethanol extract of Phellodendri chinensis cortex	Dry barks of *Phellodendron chinense* C.K.Schneid. [Rutaceae; Phellodendri chinensis cortex]	Xu et al. (2022a)
	The ethanol extract of *Lagotis brachystachya* Maxim	Dry whole *Lagotis brachystachya* Maxim	Guo et al. (2022)
	The ethanol extract of *Liriodendron chinense* (Hemsl.) Sarg	Dry peeled barks of *Liriodendron chinense* (Hemsl.) Sarg	Pan et al. (2021)
	Chinese sumac	Dry fruits of *Rhus chinensis* Mill	Ma et al. (2023)
	Polysaccharide peptide	Dry sporocarps of *Ganoderma lucidum* (Leyss.ex Fr.) Karst. [Polyporaceae; Ganoderma]	Lin et al. (2022)
	The ethanol extract of Dendrobii caulis	Dry stems of *Dendrobium nobile* Lindl [Orchidaceae; Dendrobii caulis]	Li et al. (2023a)
	The ethanol extract of *Urtica laetevirens* Maxim	Whole herbs of *Urtica laetevirens* Maxim	Han et al. (2020)
	The ethanol extract of Fraxini cortex	Branch barks of *Fraxinus rhynchophylla* Hance [Oleaceae; Fraxini cortex]	Zhou et al. (2018)
	The water extract of Eucommiae cortex	Dry Bark of *Eucommia ulmoides* Oliv [Eucommiaceae; Eucommiae cortex]	Gong et al. (2022)
	Fucoidan	The extract of *Laminariajaponica* Aresch. [Laminariaceae; Laminariae thallus eckloniae thallus]	Zhang et al. (2021c)
	The extract of Alpiniae oxyphyllae fructus	Dry ripe fruits of *Alpinia oxyphylla* Miq [Zingiberaceae; Alpiniae oxyphyllae fructus]	Lee et al. (2019)
	The ethanol extract of *Fomes igniarius* L.	Sporocarps of *Fomes igniarius* L.	Hua et al. (2023)
	The extract of *Garcinia mangostana* L.	Fruits of *Garcinia mangostana* L.	Niu et al. (2023)

In clinical practice, the core strategy for effective treatment can be considered as two aspects in early-onset and intermittent of gout: 1) in early-onset of gout: urate-lowering therapy to hyperuricemia, which relate to dissolute MSU crystals and prevent long-term of gout flares; and 2) in intermittent of gout: anti-inflammatory therapy to intermittent episodes of severely painful arthritis (gout flares) caused by deposited MSU crystals (Keller and Mandell, 2021). Thus, this review encompasses two distinct research domains: the present investigations into TCM’s impact on anti-gout molecular signaling pathways (Tables 2, 3), and the exploration of UA transporters (Table 4). The amalgamation of these findings establishes a foundational knowledge, serving as a stepping stone for the prospective creation of efficacious gout treatments.

**TABLE 2:** Regulation effect of botanical drug on signaling pathways in early-onset and intermittent of gout.

Botanical drug	Type of extract	Experiments	Animal or cell	Dose range	Model	Pos C	Neg C	Duration	Molecular mechanisms	Signal pathways	Ref.
early-onset of gout											
*Scutellaria baicalensis* Georgi (Rhizome)	Scutellarin	Po (240 mg/kg) i.p.	male C57BL/6 mice	5, 10, 20 mg/kg	*In vivo*	ALL	Saline	3 weeks	↓: UA, BUN, CRE, NLRP3, ASC, and pro-caspase-1, IL-1β, Bax, cleaved caspase-3, and cleaved caspase-9	NLRP3	Li et al. (2020b)
			HK-2 cell		*In vitro*				↑: Bcl-2, CCN1		
*Phellodendron chinense* C.K. Schneid (Bark)	Berberine	PO (300 mg/kg) i.p. and HX (300 mg/kg) i.g	male ICR mice	50, 100 mg/kg	*In vivo*	BZB	Saline	2 weeks	↓: UA, BUN, CRE, IL-18, NLRP3, ASC, Caspase1, IL-1β, URAT1	NLRP3	Zheng et al. (2023)
*Phellodendron chinense* C.K. Schneid. (Bark)	Dihydroberberine	PO (300 mg/kg) i.p. and HX (300 mg/kg) i.g	male KM mice	25, 50 mg/kg	*In vivo*	FEB	PBS	1 week	↓: UA, BUN, CRE, XOD, TNF-α, IL-1β, IL-6, IL-18, URAT1, GLUT9, NLRP3, ASC, caspase-1	NLRP3	Xu et al. (2021)
*Curcuma Longa* L. (Rhizome)	Curcumin	PO (300 mg/kg) i.p.	male KM mice	20, 40 mg/kg	*In vivo*	ALL	Vehicle	2 weeks	↓: UA, BUN, CRE, XOD, MDA, IL-1β, IL-18, NLRP3, ASC caspase1, IL-1β, MyD88, p- p65, p- IκB-α. NLRP3	NLRP3	Chen et al. (2019d)
									↑: SOD, GSH-Px		
*Polygonum cuspidatum*	Resveratrol	MSU (1 mg/40 μL) i.p	male C57BL/6 mice	15 mg/kg	*In vivo*		PBS	24 h	↓: NF-κB, P65, p-JNK, p-P38	NF-κB	Li et al. (2019b)
Siebold & Zucc (Rhizome and root)		MSU 0.2 μg/mL	THP-1 cells		*In vitro*		PBS	6 h	↑: IκBα		
*Mosla chinensis* Maxim. (Aboveground parts)	Carvacrol	PO (250 mg/kg) i.p.	male SD rats	20, 50 mg/kg	*In vivo*	ALL		1 week	↓: p-NFκB, NLRP3, TNF-α	ROS/NRLP3/NF-κB	Riaz et al. (2022)
									↑: GSH, GST, and CAT		
*Chenopodium quinoa* Willd (Fruit)	Bran Saponins	adenine (50 mg/kg) and PO (200 mg/kg) i.g.	male KM mice	25, 50, 100 mg/kg	*In vivo*			4 weeks	↓: XOD, ADA, IL-1β, TNF-α, PI3K, AKT, IKK-β, NF-κB	PI3K/AKT/NFκB	Lin et al. (2023)
		UA 200 μg/mL	HK-2 cells	100, 200, 400 μg/mL	*In vitro*			24 h			
*Coptis chinensis* Franch. (Rhizome)	Coptisine	PO (300 mg/kg) i.p and HX (300 mg/kg) i.g	male KM mice	10, 20, 40 mg/kg	*In vivo*	FEB		1 week	↓: UA, CRE, BUN SOD, ROS, MDA, TNF-α, IL-1β, IL-18 cleaved-caspase3, AIF, cleaved PARP, Bax, cyto-CytC	PI3K/Akt	Liu et al. (2022b)
		UA (20 mg/dL)	HK-2 cells		*In vitro*				↑: SOD, Bcl-2, p-Bad, p-Akt, p-PI3K		
*Gardenia jasminoides* J.Ellis (Fruit)	Geniposide	PO (300 mg/kg) i.p.	male KM mice	10, 20, 40 mg/kg	*In vivo*			2 weeks	↓: IL-1β, IL-6, TNF-α, ROS, MDA, UA, CRE, BUN	PI3K/AKT/NF-κB	Wang et al. (2022a)
									PI3K, AKT, p-NF–κB p65, Nrf2, HO-1, Keap1	Keap1/Nrf2/HO-1	
		UA (118 mg/mL)	HK-2 cells		*In vitro*				↑: SOD, TGF-β1		
*Astragalus membranaceus*. Fisch ex Bunge (Root)	Astragali Radix	PO (500 mg/kg) and adenine (200 mg/kg) i.g. and HX (300 mg/kg) i.g	male KM mice	130, 260, 520 mg/kg	*In vivo*	ALL	CMC	2 weeks	↓: XOD, ALT, AST, URAT1, GLUT9	PI3K/Akt	Zhang et al. (2023a)
									↑: ABCG2		
*Plantago depressa* Willd. (Seed)	Plantaginis Semen	PO (1.5 g/kg) i.g	male SD rats	0.9375, 1.875 3.75 g/kg	*In vivo*	BZB	Water	4 weeks	↓: UA, CRE, TG, URAT1, PI3K/Akt	PI3K/Akt	Yang et al. (2021a)
*Prunus × yedoensis* Matsum. (Aabastrum)	Naringenin	PO (250 mg/kg) and 5% fructose water i.p.	male KM mice	19, 50 mg/kg	*In vivo*	BZB	CMC	4 weeks	↓: GLUT9, URAT1, PI3K, ↑: ABCG2 P-PI3K p-AKT, AKT1	PI3K/AKT, TLR4/NF-κB	Yang et al. (2023)
		UA (8 mg/dL)	HK-2 cells		*In vitro*			12 h			
*Lonicera japonica* Thunb. (Aabastrum)	Chlorogenic acid	PO (1.5 g/kg) and adenine (0.1 g/kg)	male SD rats	40 mg/kg	*In vivo*			4 weeks	↓: UA, CRE, BUN, TNF-α, IL-6, IL-1β, ICAM-1, MCP-1 NF-κB, NF-κB p65, PI3K, AKT, mTOR	TMAO-PI3K/AKT/Mtor	Zhou et al. (2022b)
*Coptis chinensis* Franch. (Rhizome)	Berberine	PO (300 mg/kg) i.p. and HX (300 mg/kg) i.g	male KM mice	6.25, 12.5, 25 mg/kg	*In vivo*		Saline	1 week	↓: UA, CRE, BUN, GLUT9, URAT1, IL-6, JAK2, STAT3, SOCS3	JAK2/STAT3	Lin et al. (2021)
									↑: p-JAK, p-STAT3, OAT1/3, ABCG2		

*Liriodendron chinense *(Hemsl.) Sarg. (Leaf and Bark)	The exact	PO (2.4 g/kg). and adenine (150 mg/kg) i.g	male C57BL/6 mice	250, 500 mg/kg	*In vivo*	ALL	Water	3 weeks	↑: OAT1, OAT3, ABCG2	JAK2/STAT3	Pan et al. (2021)
*Genista tinctoria* L (Rhizome)	Genistein	PO (250 mg/kg) i.g.	male KM mice	10, 20 mg/kg	*In vivo*	ALL	CMC	15 days	↓: XOD, ADA, TNF-α, IL-6, MCP-1, Caspase-1, PGE2, MDA UART1, GLUT9, IL-1β, COX-2, MCP-1, mJAK2, mSTAT3 and mSOCS3 Wnt/β-Catenin ↑: SOD, CAT, GSH, T-AOC, OAT1, OAT3, OCT1, OCT2	JAK2/STAT3	Wei-Yun and Cailin (2021)
*Apium graveolens* L. (Leaf)	Apigenin	PO (300 mg/kg) i.p and HX (150 mg/kg) i.g	male KM mice	25, 50, 100 mg/kg	*In vivo*	ALL	Vehivle	1 week	↓: XOD, UA, CRE, BUN, IL-1β, IL-6, TNF-α, IL-1, IL-10, GLUT9, URAT1, SOCS3	JAK2/STAT3	Liu et al. (2022a)
									↑: P-JAK2, P-STAT3, OAT1		
*Phellodendron chinense* C.K. Schneid. (Bark)	The exact	PO (300 mg/kg) i.p. HX (300 mg/kg) i.g	male ICR mice	200, 400, 800 mg/kg	*In vivo*	FEB	Saline	10 days	↓: XOD, CRE, BUN, p-c-Jun, IL-6, MAPK3, MAPK8, AKT, TP53	MAPK	Xu et al. (2022a)
*Clerodendrum trichotomum* Thunb (Leaf)	The exact	PO (250 mg/kg) i.p.	male ICR mice	400 mg/kg	*In vivo*	COL	Saline	1 week	↓: UA, iNOS, COX-2, TNF-α	MAPK	Jang et al. (2020)
*Paeonia × suffruticosa* Andrews (Brak)	Paeonol	LPS (1 μg/mL) and MSU (200 μg/mL)	J774A.1 cells	12.5, 25, 50 μM	*In vitro*	DEX		12 h	↓: IL-1β, NLRP3	NLRP3, NF-κB, and MAPK	Chen et al. (2022)
									↑: p-IKK, p-IκBα, p-p65, p-JNK, p-ERK, p-p38		
intermittent of gout											
*Corydalis yanhusuo* W.T.Wang (Tuber)	Tetrahydropalmatine	MSU (3 mg/70 μL) i.a	male C57BL/6 mice	10, 20, 40 mg/kg	*In vivo*	COL	PBS	48 h	↓: IL-1β, IL-6, IL-18, TNF-α, NLRP3, caspase-1, MDA	NLRP3	Wang et al. (2021e)
		LPS (1 μg/mL)	RAW 264.7cell		*In vitro*			24 h	↑: GHS-Px, SOD		
*Isatis indigotica* Fortune ex Lindl. (Root)	polysaccharide	MSU (1.5 mg/50 μL) i.a	male KM mice	0.5, 1.0 g/kg	*In vivo*		PBS	24 h	↓: IL-1β, IL-18, IL-6, TNF-α, MPO, NLRP3, caspase-1, ASC	NLRP3/ASC/Caspase-1	Ren et al. (2022)
*Phellodendron amurense* Rupr. (Bark)	Palmatine	MSU (0.1 mg/20 μL) i.a	male KM mice	25, 50, 100 mg/kg	*In vivo*	COL	PBS	24 h	↓: p-65, IκBα, NLRP3, ASC, IL-1β, Caspase-1,IL-1β, IL-6, IL-18, TNF-α, MDA ↓: Nrf2, HO-1, SOD, GSH	NF-κB/NLRP3 and Nrf2	Cheng et al. (2022)
		LPS (500 ng/mL)	THP-1 cells	20, 40, 80 μM	*In vitro*			4 h			
*Rheum palmatum* L. (Rhizome and Root)	Rhein	MSU (100 μg/mL)	THP-1 cells	0.25, 0.5, 1.0	*In vitro*			24 h	↓: IL-1β, TNF- α, caspase-1, CASP1, NLRP3, ASC	NLRP3	Chang et al. (2019)
				2.5, 5.0, 10 μg/mL					↑: Nrf2, HO-1, SOD, GSH		
*Tripterygium wilfordii* Hook. f (Root)	Celastrol	MSU (1.25 mg) i.a	male WT C57BL/6J mice	0.5, 1 mg/kg	*In vivo*	MCC 905	Saline	24 h	↓:pro-caspase-1, pro-IL-1β, NLRP3, ASC	NLRP3	Yan et al. (2021)
*Salvia miltiorrhiza* Bunge. (Rhizome)	Tanshinone IIA	MSU (25 mg/mL, 0.2 mL) i.a	male SD rats	20 mg/kg	*In vivo*		Saline	6 h	↓: IL-6, TNF-α, IL-8, IL-1β, TNF-α, CXCL1, CXCL2; NLRP3, caspase-1	NLRP3	Xu et al. (2022b)
*Dioscorea polystachya* Turcz. (Rhizome)	Total saponin	MSU (400 μg/mL)	THP-1 cell	1.0, 3.0 μg/mL	*In vitro*	COL	PBS	24 h	↓: IL-1β, IL-18, TNF-α, NALP3, caspase-1 NLRP3, caspase-1, IL-18	NLRP3	Wang et al. (2020b)
*Curcuma Longa* L. (Rhizome)	Curcumin	MSU (25 mg/mL)i.a	male C57BL/6 mice	150 mg/kg	*In vivo*		PBS	6 h	↓: IL-1β, IL-6, TNF-α, COX-2, PGE2, IκBα, NF-κB, TLR4	NF-κB	Chen et al. (2019a)
		MSU 0.2 mg/mL	THP-1cell	1.0, 5.0, 10 μM	*In vitro*		PBS	24 h	MyD88, NLRP3, NF-κB		
			RAW264.7cell	5 μM	*In vitro*		PBS	24 h			

*Paeonia lactiflora* Pall. (Root)	Paeonol	MSU (60 mg/mL) i.a.	male SD rats	50, 100, 200 mg/kg	*In vivo*	COL	Saline	24 h	↓: TNF-α, IL-1β, IL-6, p65, NF-κB	NF-κB	Chen et al. (2019c)
*Paeonia lactiflora* Pall (Root)	Total glucosides of paeony	MSU 0.25 mg/mL	THP-1 cells	2.5, 5.0, 10.0 μg/mL	*In vitro*			24 h	↓: NLRP3, Caspase-1, IL-1β, MALAT1 and miR-876-5p TLR4/MyD88/NF-κB	MALAT1/miR-876-5p/NLRP3	Meng et al. (2021)
*Eugenia caryophyllata* Thunb. (Alabastrum)	β-caryophyllene	MSU (20 mg/1 mL) i.a	male SD rats		*In vivo*	IND			↓: NLRP3, Caspase-1, ASC, TLR4, MyD88, p65, IL-1β	NLRP3 and NF-κB	Li et al. (2021b)
*Cichorium intybus* L (Ground part or Root)	Cichoric Acid	MSU 1.0 mg/mL	THP-1 cells	30, 100, 300 μg/mL	*In vitro*			24 h	↓: IL-1β, TNF-α, COX-2, PGE2, NF-κB	NF-κB	Wang et al. (2021b)
									↑: IκBα		
*Epimedium brevicornu* Maxim. (Leaf)	Icariin	MSU (25 mg/mL) i.a	male SD rats	20, 40, 80 mg/kg	*In vivo*	COL			↓: IL-1β, IL-6, TNF-α, PGE2, IκB, p65, NALP3, pro-Caspase1, C-Caspase1, ASC	NF-κB/NALP3	Cao (2021)
		MSU 1000 μmol/L	Chondrocytes	1.0, 5.0, 10 μM	*In vitro*				↑: SOD, GPX, GSH		
*Lagotis brachystachya* Maxim (Grass)	The exact	MSU (50 mg/mL) i.a.	male SD rats	0.5, 1.0, 2.0 mg/kg	*In vivo*	COL	Saline	48 h	↓: UA, XOD, ALT, AST, TC, TG, ALP, AKP, MDA, TNF-α, IL-1β, IL-6, TLR4, MyD88, p-NF-κB, NLRP3, Caspase-1	TLR4/MyD88/NF-κB JAK2-STAT3 NLRP3	Guo et al. (2022)
									↑: SOD, p-JAK2, p-STAT3		
*Dioscorea polystachya* Turcz (Rhizome)	Total saponin	MSU (25 mg/mL) i.a	male Wistar rats	40, 80, 160 mg/kg	*In vivo*	COL	Saline	1 week	↓: P-38, JNK, ERK1/2, MEK1/2, MKK4, p-MKK4, ICAM1, VCAM1	MAPK and PPARs	Zhou et al. (2020)
									↑: PPARγ, p-P38, p-JNK, p-ERK1/2 p-MEK1/2		
*Rhus chinensis* Mill. (Fruit)	Chinese sumac	MSU (100 μg/mL) i.a	male SD rats	400, 800 mg/kg	*In vivo*	COL		48 h	↓: IL-1β, IL-6, TNF-α, COX-2, iNOS, NLRP3, ASC, Caspase-1 p-IκB/IκB, p-NF-κB/NF-κB, p-ERK/ERK, p-JNK/JNK, p-P38/P38	NLRP3, NF-κB, MAPK	Ma et al. (2023)

i.a., intraarticularly injected; i.p., intraperitoneally administered; i.g., intragastrically administered; s.c., subcutaneously injected; PO, potassium oxonate; HX, hypoxanthine; ALL, allopurinol; COL, colchicine; BZB, benzbromarone; FEB, febuxostat; Pos.C, positive control; Neg C, negative control.

**TABLE 3:** Regulation effect of TCM formulas on signaling pathways in early-onset and intermittent of gout.

TCM formulas	Composition	Experiments	Animal or cell	Dose range	Model	Pos C	Neg C	Duration	Molecular mechanisms	Signal pathways	Ref
Early-onset of gout.											
Simiao San (2: 2: 1: 1)	*Phellodendron chinense* C.K.Schneid. *Achyranthes bidentata* Blume. *Atractylodes lancea* (Thunb.) DC. *Coix lacryma-jobi* L.	PO (300 mg/kg) i.p. and HX (500 mg/kg) i.g	male KM mice	4.55, 9.1, 18.2 mg/kg	*In vivo*	ALL	Vehicles	1 week	↓: UA, BUN, CRE, XOD, IL-1β, IL-6, TNF-α, RAT1 GULT9, NLRP3, ASC, Cleaved-Caspase1	JAK2/STAT3 and NLRP3	Zhang et al. (2023c)
									↑: P-JAK2/JAK2, P-STAT3/STAT3, SOCS3, IL-10, OAT1		
Shiwei-Ruxiang powder (10: 15: 8: 10: 8: 12: 8: 10: 8: 5)	*Boswellia carterii* Birdw., *Terminalia chebula* Retz., *Aucklandia lappa* Decne., *Cassia obtusifolia* L., *Terminalia bellirica* (Gaertn.) Roxb., *Abelmoschus moschatus* Medik., *Phyllanthus emblica* L., *Tinospora sinensis* (Lour.) Merr., *Adhatoda vasica* *Nee*., Shilajit	PO (250 mg/kg) i.p	male C57BL/6 mice	390, 780 1,560 mg/kg	*In vivo*	ALL	CMC-Na	4 days	↓: XOD, UA, CRE BUN, TNF-α, IL-1β, p-p38 MAPK, p38 MAPK, p-IκB-α, IκB-a, p-NF-κB p65, NF-κB-p65, NLRP3, ASC, Caspase-1	MAPK and NF-κB	Li et al. (2022b)
Gegen-Qinlian decoction (8: 3: 3: 2)	*Pueraria lobata* (Willd.) Ohwi., *Coptis chinensis* Franch., *Scutellaria baicalensis* Georgi., *Glycyrrhiza uralensis* Fisch. ex DC.	MSU (3 mg/0.5 mL) i.p	male SD rats	10 mg/kg	*In vivo*			48 h	↓: URAT1, GLUT9, Bcl-2, Bax, caspase-3, caspase-8 NLRP3, TNF-α, IL-1β, IL-6, IL-8	NLRP3/GSDMD	Wang et al. (2021c)
Wuling san (3: 5: 3: 2: 3)	*Poria cocos* (Schw.) Wolf., *Polyporus umbellatus* (Pers.) Fries., *Alisma orientale* (Sam.) Juz.)., *Cinnamomum cassia* (L.) J.Presl., *Atractylodes macrocephala* Koidz.	30% fructose in drinking water	male ICR mice	987, 1316 1755, 2340 mg/kg	*In vivo*	ALL	Water	6 weeks	↓: UA, BUN, CRE, URAT1, GLUT9, TLR4 MyD88, p-IKKβ, p-IκBα, p-NF-κB, p-p38-MAPK, IL-1β, NLRP3, ASC, Caspase-1	TLR4/MyD88 and NLRP3	Yang et al. (2015)
									↑: ABCG2, OCT1, OCT2, OAT1, p-JNK, p-ERK		
Cheqianzi decoction (1: 1: 3: 1)	*Plantago asiatica* L.*Typha angustifolia* L.*Morus alba* L.*Achyranthes bidentata* Blume	adenine (100 mg/kg) i.g. and ethambutol (250 mg/kg)	male SD rats	100, 250 mg/kg	*In vivo*	ALL	CMC-Na	1 week	↓: NLRP3, IL-6, TNF-α, IL-1β, IL-18, UA, BUN, CRE, XOD	NLRP3	Meng et al. (2023)
									↑: ABCG2		
Guizhi-Shaoyao- Zhimu decoction (12: 9: 6: 12: 15: 15: 12: 12: 10)	*Cinnamomum cassia* (L.) J.Presl., *Paeonia lactiflora* Pall., *Anemarrhena asphodeloides* Bunge., *Ephedra sinica* Stapf., *Moschus berezovskii *Flerov* Atractylodes macrocephala* Koidz., *Glycyrrhiza uralensis* Fisch. ex DC., *Saposhnikovia divaricata* (Turcz. ex Ledeb.) Schischk., *Aconitum carmichaelii* Debeaux.	MSU (3 mg/mL) i.p	male C57BL/6J mice	8.4 g/kg	*In vivo*	COL	PBS	6 h	↓: IL-1β, IL-6, p-p65, IKK, p-IKK, IκB, p-IκB, NLRP3, ASC, caspase-1	NF-κB	Zhou et al. (2022a)
intermittent of gout											
Quzhuo-Tongbi (20: 10: 10: 10: 6: 4: 5: 6: 4)	*Smilax glabra* Roxb., *Dioscorea hypoglauca* Palib., *Zea mays* L., *Coix lacryma-jobi* L., *Siegesbeckiaorientalis* L., *Curcuma Longa* L., *Taxillus chinensis* (DC.) Danser., *Corydalis yanhusuo* W.T.Wang., *Citrus medica* L.	MSU (100 mg/mL) i.a	Uox-KO mice	18.0 g/kg	*In vivo*	BZB	PBS	24	↓: IL-1β, IL-6, TNF-α, IL-17	PI3K-AKT-mTOR	Song et al. (2023)
Qingre-Huazhuo - jiangsuan decoction (3: 4: 4: 6: 6: 6: 3: 3: 3: 4)	*Lonicera japonica* Thunb., *Taraxacum mongolicum* Hand.-Mazz., *Smilax glabra* Roxb., *Rheum palmatum* L., *Salvia miltiorrhiza* Bunge., *Astragalus membranaceus* Fisch. ex Bunge., *Pheretima aspergillum* (E.Perrier)., *Glycyrrhiza uralensis* Fisch. ex DC., *Achyranthes bidentata* Blume.	MSU (2.5 g/100 mL) i.a	male SD rats	1.3, 2.56, 5.15 g/mL	*In vivo*	COL	Saline	24	↓: LC3II/I, p62, ULK1, P-ULK1, Beclin-1, PI3K, AKT, mTOR	PI3K/AKT/mTOR	Liu et al. (2023)
									↑: P-PI3K, P-AKT, P-mTOR		
Wuwei-Shexiang Pills (1: 30: 30: 10: 6)	*Moschus berezovskii* Flerov., *Terminalia chebula* Retz., *Aconitum kusnezoffii* Rchb., *Aucklandia lappa* (Decne.) Decne., *Acorus calamus* L.	MSU (100 μg/20 μL) i.a	C57BL/6J mice	100, 300 mg/kg	*In vivo*	COL	Saline	24	↓: IL-1β, TNF-α, NLRP3, Caspase-1	NF-κB and MAPK	Bai et al. (2023)
Shizhifang	*Malva verticillata Plantago asiatica *L*.Sinapis alba *L*.Vaccaria segetalis (Neck.)* Garcke ex Asch	PO (250 mg/kg) i.p.	BALB/c mice	11.25 g/kg	*In vivo*	ALL		2 weeks	↓: IL-1β, IL-18, NLRP3, pro-caspase-1, caspase-1	NLRP3//GSDMD	Zhou et al. (2023)
		UA (200 g/mL)	HK-2 cell		*In vitro*				GSDMD, N-GSDMD		
Fufang-Zhenzhu-Tiaozhi capsule (15: 10: 5: 5: 5: 5: 5: 3)	*Ligustrum lucidum *W.T.Aiton *Cirsium japonicum *DC*. Atractylodes macrocephala *Koidz*. Salvia miltiorrhiza *Bunge*. Eucommia ulmoides *Oliv*. Citrus medica *L*. Panax notoginseng *(Burkill.) F. H*. Chen Coptis chinensis *Franch**	HX (300 mg/kg) and PO (500 mg/kg) i.g.	male Balb/c mice	600, 1200 mg/kg	*In vivo*	ALL BZB	CMC-Na	3 weeks	↓: UA, CRE, BUN, NLRP3, IL-1β, GULT9, PI3K, AKT	PI3K/AKT/NF-κB	Li et al. (2022a)
Modified Baihu decoction (24: 4: 4: 3: 4: 3: 6: 2: 6: 6: 4: 4)	*Gypsum Fibrosum* Anemarrhena asphodeloides *Bunge*. Paeonia lactiflora *Pall*. Achyranthes bidentata *Blume* *Corydalis yanhusuo* W.T.Wang *Phellodendron amurense* Rupr. *Atractylodes lancea* (Thunb.) DC. *Glycyrrhiza inflata* Batalin *Coix lacryma-jobi* L., *Lonicera japonica* Thunb., *Reynoutria japonica* Houtt., *Clematis chinensis* Osbeck	MSU (50 mg/mL) i.a.	male SD rats	5.85, 35 g/kg	*In vivo*	IV	Water	3 weeks	↓: TNF-α, IL-1β, NLRP3, ASC, Caspase-1	NLRP3	Wang et al. (2022b)
Simiao decoction (2: 2: 1: 1)	*Phellodendron chinense *C.K.Schneid*. Achyranthes bidentata *Blume*. Atractylodes lancea (*Thunb.) DC*. Coix lacryma-jobi *L*.*	MSU (1.0 mg/40 μL) i.a.	male C57BL/6 mice	4, 8, 16 mg/kg	*In vivo*	FEB	Saline	48 h	↓: MPO, XOD, ADA, UA IL-1β, IL-9, IFN-γ, MIP-1α, MIP-1β	NLRP3	Lin et al. (2020a)
Simiao san (2: 2: 1: 1)	*Phellodendron chinense *C.K.Schneid*. Achyranthes bidentata *Blume*. Atractylodes lancea *(Thunb.) DC*. Coix lacryma-jobi *L*.*	MSU (1 mg/50 μL) i.a.	male C57BL/6 mice	1, 10 mg/kg	*In vivo*	Saline	Saline	3 weeks 24h	↓: NLRP3, IL-6, IL-1β	PI3K/Akt	Cao et al. (2021)

i.a., intraarticularly injected; i.p., intraperitoneally administered; i.g., intragastrically administered; s.c., subcutaneously injected; PO, potassium oxonate; HX, hypoxanthine, ALL, allopurinol; COL: colchicine; BZB, benzbromarone; FEB, febuxostat; Pos.C, positive control; Neg C, negative control.

**TABLE 4:** Regulation effect of botanical drug on uric acid transporters in early-onset of gout.

Botanical drug	Type of extract	Experiments	Animal or cell	Dose range	Model	Pos C	Neg C	Duration	Molecular mechanisms	Ref
*Dioscorea hypoglauca* Palib (Rhizome)	Dioscin	adenine (75 mg/kg) and PO (200 mg/kg) i.g	male KM mice	25, 50 mg/mL	*In vivo*	BZB	acacia water	2 weeks	↓: URAT1, GLUT9	Zhou et al. (2018)
					*In vitro*				↑: ABCG2, OAT1	
*Eurycoma longifolia* Jack (Stem)	Eurycomanol	adenine (75 mg/kg) and PO (200 mg/kg) i.g	C57BL/6 J mcie	5, 10, 20 mg/kg	*In vivo*	BZB	acacia water	1 week	↓: XOD, UA, CRE, BUN, GLUT9	Bao et al. (2022)
									↑: ABCG2, OAT1, NPT1	
*Bergenia crassifolia* (L.) Fritsch (Grass)	Bergenin	PO (250 mg/kg) andhigh-glucose i.p	male C57BL/6 mice	40, 80 mg/kg	*In vivo*	ALL	Saline	3 weeks	↓: UA, XO, IL-6, IL-1β, TNF-α, GLUT9	Chen et al. (2020)
									↑: ABCG2	
*Smilax glabra* Roxb (Rhizome)	Total Flavonoids	PO (250 mg/kg) i.g	male KM mice	62.5, 125, 250 mg/kg	*In vivo*	ALL	CMC-Na	1 week	↓: UA, CRE, BUN, XOD	Huang et al. (2019)
									↑: OAT1, OCTN2	
*Nelumbo nucifera* Gaertn. (Leaf)	Nuciferine	PO (250 mg/kg) and adenine (100 mg/kg) i.p	male KM mice	10, 20, 40 mg/kg	*In vivo*	BZB	CMC-Na	2 weeks	↓: URAT1, GLUT9	Zhang et al. (2022b)
		UA 8 mg/dL	HK-2 cells	10, 20, 40 μM	*In vitro*			24 h	IL-18, TXNIP, ASC, caspase-1, NLRP3, GLUT9, URAT1	
*Phellodendron chinense* C.K.Schneid. (Bark)	Palmatine	PO (300 mg/kg) and HX (300 mg/kg) i.g	male KM mice	25, 50, 100 mg/kg	*In vivo*	FEB	CMC-Na	1 week	↓: UA, CRE and BUN, XOD, ADA, MDA, IL-1β	Ai et al. (2023)
*Lagotis Brachystachya* Maxim. (Grass)	Luteolin and Luteoloside	PO (350 mg/kg) i.p	male KM mice	20, 50 mg/kg	*In vivo*	ALL	Vehicle	1 week	↓: IL-1β, TNF-α, TLR4, NLRP3, MyD88, GLUT9, URAT1	Zhu et al. (2021)
		UA (200 g/mL)	HK-2 cells	3.125,6.25 μM	*In vitro*	BZB		24 h	↑: OAT1	
*Caragana sinica* (Buc’hoz) Rehd (Flower)	α-viniferin	PO (100 mg/kg) i.p HX (500 mg/kg) i.g	male KM mice	10, 20, 40 mg/kg	*In vivo*	ALL	Saline	1 week	↓: IL-17, URAT1, GLUT9	Guo et al. (2023)
									↑: ABCG2, OAT1	
*Moringa Oleifera* Lam. (Bark)	Flavonoids and Phenolics	PO (300 mg/kg) i.p	male KM mice	1200, 2400 mg/kg	*In vivo*	ALL	Saline	1 week	↓: URAT1, GLUT9	Luo et al. (2022)
		HX (300 mg/kg) i.g							↑: ABCG2, OAT1, OAT3	
*Ganoderma lucidum*	Polysaccharide	PO (300 mg/kg) and	male ICR mice	50 mg/kg	*In vivo*	ALL	Saline	2 weeks	↓: GLUT9 ↑: OAT1	Lin et al. (2022)
(Leyss.ex Fr.) Karst. (Sporocarp)	peptide	HX (300 mg/kg) i.p	HK-2 cells	6.25, 25, 100 μg/mL	*In vitro*	ALL				
*Poria cocos* (Schw.) Wolf (Sclerotium)		PO (300 mg/kg) i.p. and	male KM mice	50, 100, 200 mg/mL	*In vivo*	ALL	Saline	1 week	↓: UA, BUN, CRE, ALT, AST, ALP	Liang et al. (2021)
		HX (500 mg/kg) i.g							↑: ABCG2	

*Dendrobium nobile* Lindl. (Stem)	Ethanol extract	PO (200 mg/kg) and high-sugar and high-fat diet and Red Star Erguotou i.g	male SD rats	0.75, 1.5 3.0 mg/kg	*In vivo*	BZB	Saline	12 weeks	↓: UA, CRE, AST, ALT, ADA, XOD, IL-6, IL-8, TNF-α, GLUT9	Li et al. (2023a)
*Urtica laetevirens* Maxim. (Grass)	Ethanol extract	PO (350 mg/kg) i.p	male KM mice	25, 50, 100 mg/kg	*In vivo*	ALL	Saline	1 week	↓: CRE, BUN, XOD, ADA, URAT1	Han et al. (2020)
			HK-2 cells	0.78, 2.34 μg/mL	*In vitro*			24 h	↑: OAT1	
*Fraxinus rhynchophylla* Hance (Bark)	Fraxini Cortex	PO (250 mg/kg) i.p. and HX (10 mg/kg) s.c	male SD rats	50, 100, 200 mg/kg	*In vivo*	ALL	Saline	2 weeks	↓: UA, CRE, URAT1, GLUT9	Zhou et al. (2018)
*Eucommia ulmoides* Oliv. (Leaf)	Water extract	High-fat and 20% diet fructose wate	male Wistar rats	100, 200 mg/kg	*In vivo*		CMC-Na	8 weeks	↓: IL-6, TNF-α, TLR4, GLUT9	Gong et al. (2022)
*Laminariajaponica* Aresch. (Leaf)	Fucoidan	UA (600 μg/mL)	HK-2 cells	100, 200 μg/mL	*In vitro*			24 h	↓: p65, JNK, AKT, GLUT9, URAT1	Zhang et al. (2021c)
									↑: p-p65, p-JNK, p-AKT	
*Chrysanthemum Indicum* L. (Flower) and *Cornus officinalis* Siebold & Zucc.(Fruit)	combination extract	PO (200 mg/kg) i.p	C57BL/6J mice	150, 300, 600 mg/kg	*In vivo*	ALL	Saline	1 week	↓: URAT1, GLUT9	Kim et al. (2021)
									↑: OAT1, OAT3	
*Alpinia oxyphylla* Miq. (Fruit)	The extract	PO (150 mg/kg) i.p	male SD rats	200, 400 mg/mL	*In vivo*	BZB	CMC-Na	5 days	↓: GLUT9	Lee et al. (2019)
									↑: OAT1	
*Cichorium intybus* L (Grass)	The extract	PO (250 mg/kg) i.p	male SD rats	100, 300, 500 mg/kg	*In vivo*	ALL	Water	5 days	↓: URAT1, GLUT9	Jeong et al. (2022)
									↑: ABCG2, OAT1, OAT3	
*Fomes igniarius* L	Ethanol extract	PO (250 mg/kg) and	male ICR mice	125, 250, 500 mg/kg	*In vivo*	ALL	CMC-Na	3 weeks	↓: URAT1, GLUT9	Hua et al. (2023)
(Sporocarp)		adenine (100 mg/kg) i.g							↑: ABCG2, OAT1, OAT3	
*Camellia sinensis* (L.) Kuntze	Epigallocatechin	PO (200 mg/kg) s.c and ethambutol (250 mg/kg) i.g	male SD rats	25, 50, 100 mg/kg	*In vivo*	ALL		1 week	↓: UA, CRE, BUN, XOD, GLUT9	Li et al. (2020a)
									↑: OAT1	
*Garcinia mangostana *L	α-Mangostin	PO (500 mg/kg) i.g	male KM mice	5, 10, 20 mg/kg	*In vivo*	ALL	CMC-Na	1 week	↓: URAT1, GLUT9	Niu et al. (2023)
									↑: SOD, CAT, GSH, ABCG2, OAT1	

*Citrus aurantium* L. and	Hesperetin	PO (450 mg/kg) i.p	male ICR mice	5, 10 mg/kg	*In vivo*	ALL	Vehicle	2 weeks	↓: MDA, FOXO3a, IL-18, TLR4, NF-κB, NLRP3	An et al. (2023)
*Citrus sinensis* Osbeck (fruit)			HEK293T cells		*In vitro*				↑: GSH-PX, CAT, OAT1, OAT3, OCT2	
*Prunus mandshurica*	Phloretin	adenine (50 mg/kg) and	male C57BL/6 mice	50 mg/kg	*In vivo*		CMC-Na	3 weeks	↓: XOD, IL-1β, IL-18, caspase-1	Cui et al. (2020)
(Maxim.) Koehne		PO (1 g/kg) i.g	HK-2 cells		*In vitro*				NLRP3, ROS, GLUT9, URAT1	
*Sambucus canadensis* L. (fruit)	Anthocyanins	PO (280 mg/kg) and inosine (400 mg/kg) i.p	male ICR mice	400, 800 mg/kg	*In vivo*	ALL	CMC-Na	1 week	↓: XOD, caspase-1, TNF-α IL-1β, URAT1, GLUT9	Qian et al. (2019)
									↑: ABCG2, OAT1, OAT3, OCT1, OCT2 OCTN1, OCTN2	
*Lonicera japonica* Thunb (Aabastrum)	Chlorogenic acid	PO (300 mg/kg) i.p. and HX (300 mg/kg) i.g	male KM mice	40 mg/kg	*In vivo*	ALL	CMC-Na	3 weeks	↓: XOD, UA, CRE, BUN, IL-1β, TNF-α, caspase-1, NLRP3, TLR4, NF-κB, GLUT9, URAT1	Zhou et al. (2021)
									↑: ABCG2, OAT1	
*Mangifera indica* L (fruit)	Mangiferin	HX (300 mg/kg) i.g. and PO (300 mg/kg) s.c	male ICR mice	50 mg/kg	*In vivo*		CMC-Na	10 days	↓: COX-2, PGE2, NO, IFN-γ, URAT1, GLUT9	Li et al. (2020d)
									↑: ABCG2	
the exact of honey, propolis, and mushrooms	Chrysin	given a 10% high-fructose syrup solution	male SD rats	50, 100, 150 mg/kg	*In vivo*	ALL	CMC-Na	12 weeks	↓: XOD, MDA, IL-1β, ASC, caspase-1 NLRP3, URAT1, GLUT9	Chang et al. (2021)
									↑: OAT1, ABCG2	
*Scutellaria baicalensis* Georgi (Root)	Baicalein	PO (300 mg/kg) i.p	male KM mice	100, 200 mg/kg	*In vivo*	ALL	CMC-Na	2 weeks	↓: XOD, URAT1, GLUT9	Chen et al. (2021)
*Veratrum grandiflorum* (Maxim. ex Miq.) O.Loes(root)	Resveratrol	given a high-fat diet	C57BL/6J mice	100 mg/kg	*In vivo*		Vehicle	12 weeks	↓: XOD, IL-6, IL-18, IL-1β, TNF-α, URAT1, GLUT9	Zhang et al. (2021b)
*Fraxinus rhynchophylla* Hance (Branch bark)	Esculetin	HX (300 mg/kg) and PO (300 mg/kg) i.p	male KM mice	33.3 100, 300 mg/kg	*In vivo*	BZB	CMC-Na	2 weeks	↓: XOD, URAT1, GLUT9	Wang et al. (2020c)
			HK-2 cells		*In vitro*				↑: SOD, GSH, GPx, OAT1, OAT3	

i.a., intraarticularly injected; i.p., intraperitoneally administered; i.g., intragastrically administered; s.c., subcutaneously injected; PO, potassium oxonate; HX, hypoxanthine, ALL, allopurinol; COL, colchicine; BZB, benzbromarone; Pos.C,:positive control; Neg C, negative control.

## 2 Signal pathways of TCM modulating gout pathological progression

### 2.1 NLRP3 signal pathway

One of the most extensively studied and vital inflammasomes is the NOD-like receptor thermal protein domain-associated protein 3 (NLRP3). This vital component comprises the sensing molecule NLRP3, coupled with apoptosis-associated speck-like (ASC) adaptors, caspase activating recruitment domain, and recruitment caspase-1. Activation of nuclear factor κ-light-chain-enhancer of activated B cells (NF-κB) prompts the upregulation of NLRP3 expression. Following this, it combines and forms an intricate configuration with an adapting protein, ASC, and procaspase-1. Caspase-1 is subsequently activated through the autolysis of procaspase-1. This sequential process subsequently triggers the handling and liberation of interleukin (IL)-1β, IL-18, along with alarm signals. Additionally, it leads to the initiation of pyroptotic cell demise (Yang et al., 2019; de Almeida et al., 2022). Pathogen-associated molecular patterns (PAMPs), including bacterial toxins, microorganisms, and lipopolysaccharides (LPS), contribute to the activation of NLRP3. Additionally, NLRP3 displays responsiveness to various damage-associated molecular patterns (DAMPs), such as MSU, alum, silica, ATP, and others (Biasizzo and Kopitar-Jerala, 2020). Since NLRP3 recognizes a wide range of PAMPs and DAMPs, it is often involved in the pathogenesis of various human diseases as a central sensor of these inflammatory conditions (Zhan et al., 2022). The NLRP3 inflammasome has been established as a primary mechanism by which MSU crystals initiate the inflammatory response (So and Martinon, 2017) through the assembly of NLRP3 and the activation of caspase-1 (Goldberg et al., 2017). Activation of IL-1β triggers the production of IL-6, IL-18, and tumor necrosis factor-α (TNF-α), prompting the migration of neutrophils and other cells into the synovial fluid and synovium, ultimately inciting the onset of inflammation (Hu et al., 2019). Research has demonstrated an elevation in the expression of NLRP3, ASC, IL-1β, and Caspase-1 in joint tissues during the progression of gout (Lin et al., 2020b). Concurrently, additional researchers have also investigated how the inflammatory response initiated by MSU crystals decreases due to the decrease in proteins linked with NLRP3, consequently alleviating both pain and swelling (Yin et al., 2020; Wang et al., 2021a).

Scutellarin is a bioactive flavonoid extracted from the traditional Chinese botanical drug *Scutellaria baicalensis* Georgi. This compound exhibits neuroprotective, antioxidant, anti-inflammatory, and anti-tumor properties (Chen et al., 2015; Baluchnejadmojarad et al., 2018; Li et al., 2019a). It has the capacity to notably decrease the expression of cleaved-Caspase-3, cleaved-Caspase-9, protein B-cell lymphoma-2 (Bcl-2) associated X protein (BAX), while elevating the levels of the antiapoptotic Bcl-2. Furthermore, scutellarin mitigates the expression of proteins linked to the NLRP3 pathway (NLRP3, ASC, Caspase1, and IL-1β). Through its modulation of the NLRP3 signaling pathway, scutellarin can enhance renal damage caused by UA (Li et al., 2020b). Tetrahydropalmatine, a primary bioactive compound with analgesic, anti-addictive, anti-inflammatory, neuroprotection, and anticancer activities (Du et al., 2022), found in *Corydalis yanhusuo* (Y.H.Chou & Chun C.Hsu) W.T.Wang, has demonstrated a noteworthy ability to reduce the levels of NLRP3, caspase-1, and IL-1β in the paw tissues of mice with MSU-induced acute gout. It also diminishes proinflammatory cytokines such as TNF-α, IL-1β, IL-6, and IL-18, and thereby alleviating gout-related pain and swelling. This compound acts therapeutically by inhibiting the activation of the NLRP3 inflammasome and the subsequent caspase-1 formation (Wang et al., 2021e). The utilization of *Isatis indigotica* Fortune ex Lindl. polysaccharide (can lead to a reduction in the levels of NLRP3, ASC, and caspase-1. This modulation aids in alleviating symptoms linked to MSU-induced gouty arthritis, such as neutrophil infiltration in knee joints, joint circumference and the production of TNF-α, IL-1β, IL-6, and IL-18. This effective inhibition of the NLRP3 signaling pathway is facilitated by this approach (Ren et al., 2022). Furthermore, palmatine (Cheng et al., 2022), rhein (Chang et al., 2019), berberine (Zheng et al., 2023), dihydroberberine (Xu et al., 2021), celastrol (Yan et al., 2021), tanshinone IIA (Xu et al., 2022b), total saponin of *Dioscorea polystachya* Turcz. (Wang et al., 2020b), isoorientin (An et al., 2021), urcumin (Li et al., 2019c) and curcumin (Chen et al., 2019d) have been found to possess anti-inflammatory properties by impeding the NLRP3 signaling pathway. Consequently, these compounds contribute to the improvement of gout-associated conditions.

In a study by Lin et al. (2020a), it was revealed that Simiao decoction effectively eased gouty arthritis symptoms in mice. This decoction led to a decrease the levels of IL-1β, IL-6, and TNF-a within colon tissue, and it hindered the growth of harmful gut bacteria while also suppressing the levels of Caspase-1, ASC, and NLRP3. These observations suggest that Simiao decoction has the potential to ease gouty arthritis by blocking the NLRP3 signaling pathway and opposing inflammation in the gastrointestinal tract. An additional study demonstrated a reduction in miRNA levels of Bax, caspase-3, caspase-8, and Bcl-2 within the renal tissue of hyperuricemic rats treated with Gegen-Qinlian decoction. Furthermore, it decreased the expression of Bcl-2 and caspase gene family proteins in an *in vitro* UA-stimulated model. These results indicate that Gegen-Qinlian decoction could potentially serve as a favorable approach for gout treatment, functioning by inhibiting apoptosis and renal inflammation (Wang et al., 2021c).

Furthermore, the Chinese Formula Wuling san (Yang et al., 2015), Cheqianzi decoction (Meng et al., 2023), Baihu decoction (Wang et al., 2022b), Sanhuang- Xiexin decoction (Wu et al., 2019) and Shizhi fang (Zhou et al., 2023) have exhibited significant effects in gout prevention by operating through pathways linked to the inhibition of the NLRP3 signaling cascade and the mitigation of renal damage.

### 2.2 NF-κB signal pathway

Recognized as the key intermediary in inflammation and energy insufficiency, NF-κB governs the synthesis of pro-inflammatory cytokines (Saito and Tanaka, 2017). The family of NF-κB transcription factors encompasses five structurally akin proteins: p50, p52, p65, RelB, and c-Rel (Zhang et al., 2019a). IκBα, which serves as the suppressor of NF-κB, is regulated by the IκB kinase (IKK) complex. Upon stimulation, the NF-κB pathway is activated by degradation of the IκB or ubiquitination of p100 (Mitchell et al., 2016), subsequently orchestrating an inflammatory role by governing the expression of downstream genes, including proinflammatory cytokines (TNF-α, IL-1, and IL-6) (Lepetsos et al., 2019). Studies have indicated that MSU crystals facilitate the nuclear translocation of proteins associated with the NF-κB signaling cascade and the expression of inflammatory factors, finally exacerbating the severity of gout (Chen et al., 2019a; Cao, 2021).

Obtained from *Paeonia × suffruticosa* Andrews, paeonol exhibits anti-inflammatory characteristics by decreasing the concentrations of proinflammatory cytokines (TNF-α, IL-1β, and IL-6) and reducing p65 within the nucleus. Additionally, it lessens NF-κB DNA-binding activity in synovial tissues of rats experiencing inflammation induced by MSU (Chen et al., 2019c). Icariin, an active compound derived from the botanical drug *Epimedium brevicornu* Maxim. has demonstrated the ability to diminish inflammation and safeguard the functionality of ankle joints. This is achieved by decreasing the levels of TNF-α, IL-1β, and IL-6, along with Prostaglandin E2 (PGE2). Furthermore, it inhibits the nuclear translocation of p65 and IκB, thus effectively mitigating the symptoms of acute gout arthritis (Cao, 2021). Curcumin, the primary bioactive compound in *Curcuma Longa* L. hinders the degradation of IκBα and phosphorylation of NF-κB subunits (p50 and p65). This dual effect leads to decreased expression of pro-inflammatory cytokines such as TNF-α, IL-1β, and IL-6, as well as cyclooxygenase-2 (COX-2) and PGE2. As a consequence, a protective impact of curcumin is exerted against gout by alleviating the inflammatory response via modulation of the NF-κB signaling pathway (Chen et al., 2019a). The active biological component found in the roots of *Paeonia lactiflora* Pall., known as total glucosides of *P. lactiflora* Pall. demonstrated the ability to lower the expression of toll-like receptor 4 (TLR4), myeloid differentiation factor 88 (MyD88), as well as several key proteins involved in the NF-κB pathway, including phospho-IKBα, IKBα, phospho-p65, and p65. This led to the inhibition of the TLR4/MyD88/NF-κB pathway’s activation triggered by MSU, ultimately providing relief from gout symptoms (Meng et al., 2021). Furthermore, isovitexin (Sun et al., 2021), β-caryophyllene (Li et al., 2021b), cichoric acid (Wang et al., 2021b), resveratrol (Li et al., 2019b), carvacrol (Riaz et al., 2022) and bran saponins (Lin et al., 2023) can offer a safeguarding impact against gout by suppressing the NF-κB signaling pathway.

Several TCM formulations have been observed to decelerate the progression of gout. For instance, Guizhi-Shaoyao-Zhimu decoction, traditionally utilized in TCM for managing “Bi Zheng” exhibited the ability to diminish neutrophil recruitment and lower the concentrations of IL-6, IL-1β, and monocyte chemoattractant protein-1 (MCP-1) (Zhang et al., 2019b). It also hindered the activation of NF-κB signaling pathways by reducing p-IKKβ and p-p65 levels, augmenting IκBα levels, and mitigating NF-κB’s binding to DNA (Wei et al., 2021). Consequently, this formulation effectively shielded mice induced by MSU from inflammation (Zhou et al., 2022a). Moreover, adapted Sanmiao pills, widely utilized in China for the management of gouty arthritis, have demonstrated capability to mitigate the harm caused to ankle joints by MSU and hinder the release of inflammatory cytokines (such as TNF-α, IL-6, and IL-1β), alongside reducing the levels of pivotal proteins engaged in the TLRs/MyD88/NF-κB signaling pathway (Chen et al., 2023).

### 2.3 PI3K/AKT signal pathway

Phosphatidylinositol 3 kinase (PI3K) and protein kinase B (AKT) are upstream elements of the NF-κB signaling cascade, pivotal in the initiation and release of various inflammatory factors (Wang et al., 2020a). Elevated levels of UA have the capacity to attach to receptors situated on the cell membrane, engaging with receptors present on the membrane’s surface. This interaction triggers the activation of PI3K and AKT, thereby initiating a sequence of subsequent reactions in mice with hyperuricemia (Wang et al., 2022a). Moreover, the process of autophagy is governed by PI3K/AKT pathway andmammalian target of rapamycin (mTOR) (Evangelisti et al., 2020), and it is implicated in the inflammatory response of gouty arthritis (Lou et al., 2022). The MSU crystal has also been found to activate autophagy with subsequent cell death by inhibiting the phosphorylation of the AKT/mTOR signaling cascade (Hwang et al., 2015), thereby playing a crucial role in the promotion of gout (Xiao et al., 2023). PI3K enzymes are grouped into three primary categories (Class I, II, and III) according to their structural characteristics and substrate specificity. Among these, PI3KI, which contributes to various biological activities, has garnered the most research attention (Guo et al., 2015). Phosphoinositide-associated kinase-1 (PDK1) and AKT are brought to the inner layer of the cellular membrane via pleckstrin homology regions. AKT reveals two principals phosphorylation sites (Thr308 and Ser473), which are phosphorylated by PDK1 and mTORC2, respectively (Manning and Toker, 2017). Following full activation, AKT is orchestrating distinct subsequent cascades and carrying out diverse functions like governing cell viability, metabolism, and growth by impacting the activation statuses of numerous downstream effectors (Chen et al., 2018).

Coptisine, a prominent bioactive compound found in *Coptis chinensis* Franch. diminishes the levels of cleaved poly ADP-ribose polymerase (PARP), cleaved-caspase-3, Bax, cytochrome C (cytC), and apoptosis-inducing factor (AIF). Concurrently, it elevates the protein levels of Bcl-2 and p-Bad, thereby offering protection against oxidative stress, mitochondrialapoptosis, and renal inflammatory harm triggered by hyperuricemia. This safeguarding effect is achieved by modulating the PI3K/AKT signaling pathway (Liu et al., 2022b). From the fruit of *Gardenia jasminoides* J.Ellis., it was found that an iridoid glycoside, called geniposide, can be extracted, which has antiarthritic properties (Wang et al., 2021d; Wang et al., 2022c; Ke et al., 2022). The geniposide-phospholipid complex reduces levels of inflammatory cytokines (TNF-α, IL-6, and IL-1β) in hyperuricemic mice and regulates the PI3K/AKT/NF-κB signaling cascade to improve post-hyperuricemia chronic kidney disease (Wang et al., 2022a). Naringenin, a flavonoid found in citrus fruits known for its diverse biological effects, offers renal protection by reducing elevated IL-6 and TNF-α level. Additionally, it improves hyperuricemia conditions by boosting UA excretion through the inhibition of the PI3K/AKT signaling cascade in the kidney (Yang et al., 2023). Chlorogenic acid, present in ample amounts within *Lonicera japonica* Thunb. and *Eucommia ulmoides* Oliv., has been observed to lower the protein levels of phosphorylated AKT (P-AKT), PI3K, and mTOR. It effectively safeguards against hyperuricemia-induced kidney damage by activating the PI3K/AKT signaling cascade (Zhou et al., 2022b).

Gout falls under the “arthralgia syndrome” classification in TCM, arising from the accumulation of turbid toxins, heat, andblood stasis. A formulation known as Qingre-Huazhuo-Jiangsuan decoction, designed to eliminate turbidity, dispel heat, decrease acidity, and combat inflammation, exhibits the ability to mitigate the inflammatory state of acute gouty arthritis (wang et al., 2018). This is achieved by activating autophagy genes and suppressing the PI3K/AKT/mTOR pathway in knee synovial tissue (Liu et al., 2023). Furthermore, Quzhuo-Tongbi decoction effectively enhances the presence of allobaculum and candidatussaccharimonas in the gut microbiome, decrease concentration of inflammatory cytokines (TNF-α, IL-6, IL-17, and IL-1β), and restores the equilibrium between Th17 and Treg cells. This comprehensive approach regulates the differentiation of CD4^+^ T cells through the PI3K/AKT/mTOR cascade and remodels the gut microbiome, offering a strategy for gout treatment (Song et al., 2023).

Furthermore, certain herbal remedies have demonstrated gout-protective effects, including Huangqin-Qingre-Chubi capsule (Zhang et al., 2023b), Fufang -Zhenzhu-Tiaozhi capsule (Li et al., 2022a) and Simiao san (Cao et al., 2021), and some herbal medicines, such as *Astragalus membranaceus* Fisch. ex Bunge (Zhang et al., 2023a), *Phellodendron chinense* C.K.Schneid (Xu et al., 2022a). and *Plantago depressa* Willd (Yang et al., 2021a). These interventions have been linked to anti-inflammatory effects and the modulation of the PI3K/AKT signaling pathway.

### 2.4 JAK/STAT signal pathway

The Janus Kinase (JAK), and the signal transducer and activator of transcription (STAT) signaling pathway is composed of tyrosine kinase-related receptors called JAK, and STAT (Li et al., 2015), This pathway is interconnected with various bodily functions and is involved in significant biological processes such as immune regulation, cell proliferation, apoptosis, and differentiation (Xin et al., 2020). STAT assumes a critical role in both signal activation and transcription. In the cytoplasmic, the STAT group functions as a downstream recipient for JAKs, which are intracellular tyrosine kinases linked to the internal domains of receptors situated on the cell membrane (Chapman et al., 2022). In a general sense, external signals like IL-2 to IL-7, epidermal growth factor, and interferon (IFN) (O'Shea et al., 2015) attach to their respective receptors located on the cell membrane. This interaction leads to the proximity of receptor-associated JAK kinases, resulting in their mutual phosphorylation and activation. Subsequently, the activated receptors phosphorylate STAT proteins, prompting their migration from the inner cellular surroundings to the nucleus (Morris et al., 2018), Once in the nucleus, these STAT proteins bind to DNA and orchestrate the regulation of gene transcription. Depending on the tissue specificity and the receptors engaged in the signaling process, diverse JAKs and STATs become enlisted in this mechanism (Savage et al., 2020), which exhibits a strong connection with the onset and progression of different diseases (Zhang et al., 2022a). During gout flare-ups, the activation of JAK2/STAT3 signaling results in an elevation of cytokine expression, including IL-6, IL-1β, and TNF-α, within the kidneys and joints. Consequently, this provokes an inflammatory reaction towards the accumulation of crystals (Xin et al., 2020; Ren et al., 2021). Simultaneously, the reduction of the JAK/STAT signaling cascade can curtail the deposition of monosodium urate crystals and lower serum urate levels, effectively suppressing joint pain and inflammation (Cunningham et al., 2016).

Berberine, the primary constituent of *Phellodendron chinense* C.K.Schneid., has exhibited the capacity to diminish the expression of phospho-JAK2, phospho -STAT3, and suppressor of cytokine signaling 3 (SOCS3). Moreover, it lessens renal concentrations of TNF-α, IL-1β, and IL-6 in mouse models of hyperuricemia, thereby mitigating renal impairment. This is achieved through the inhibition of the JAK-STAT signaling pathway’s activation and the attenuation of the inflammatory response triggered by UA (Lin et al., 2021). Apigenin, a plant-derived flavone compound, is acknowledged as a biologically active flavonoid known for its anti-inflammatory properties. It exhibits a robust affinity for JAK2 proteins and, UA transporters effectively enhancing UA metabolism and mitigating renal damage (Liu et al., 2017b). This is accomplished by curtailing UA production, enhancing excretion, and dampening the activity of the JAK2/STAT3 signaling pathway in hyperuricemia mice (Liu et al., 2022a). *Lagotis brachystachya* Maxim., primarily employed for treating yellow water disease (gouty arthritis) and hepatitis, is capable of reducing the expression of phospho-JAK2 and phospho-STAT3. It also operates to hinder the JAK2/STAT3 signaling cascade within synovial tissues, thereby alleviating ankle joint swelling and gout-induced inflammation in rats (Guo et al., 2022). *Liriodendron chinense* (Hemsl.) Sarg., a component of TCM employed to expel “wind and dampness”, has been observed to impede the advancement of hyperuricemic nephropathy. This is achieved by curbing the activation of the JAK2/STAT3 signaling cascade, lessening the influx of inflammatory agents, and diminishing the buildup of UA within the kidney (Pan et al., 2021). Genistein, an isoflavone initially extracted from the flowering plant *Genista tinctoria* L., has been identified for its ability to diminish the levels of renal mJAK2, mSTAT3, and mSOCS3. This action leads to a reduction in renal inflammation and show cases a safeguarding effect against potassium oxonate-induced hyperuricemia in mice (Wei-Yun and Cailin, 2021).

Furthermore, both *in vivo* and *in vitro* studies have demonstrated that Simiao wan can effectively lower the concentrations of TNF-α, IL-6, and IL-1β. It achieves this by suppressing the expressions of phospho-JAK2/JAK2, phospho-STAT3/STAT3, and SOCS3, thereby constraining the activity of the JAK2/STAT3 signaling pathways. This comprehensive approach contributes to the alleviation of hyperuricemia and renal inflammation (Yang et al., 2021b; Zhang et al., 2023c).

### 2.5 MAPK signal pathway

Mitogen-activated protein kinases (MAPKs), comprising extracellular signal-regulated kinases (ERK1/2), Jun N-terminal kinase (JNK), and P38, play crucial roles in the onset of gouty arthritis episodes. Serving as precursor kinases, mitogen-activated protein kinase kinase 4/7 (MKK4/7), MKK3/6 andmitogen-activated extracellular signal-regulated kinase 1/2 (MEK1/2) govern the activation of JNK, P38, and ERK1/2, respectively (Zhang et al., 2018b; Stramucci et al., 2018; Wang et al., 2019). Recent studies have indicated that UA has the potential to induce the promotion and migration of vascular smooth muscle cells (VSMCs) through the activation of the p38 and p44/42 MAPK pathways in individuals with hyperuricemia (Doğru et al., 2022), Ultimately, this process contributes to vascular remodeling and the development of other associated conditions (Canepa et al., 2017; Oğuz et al., 2017). MAPKs, which form part of an extensive group of serine-threonine kinases, create signaling pathways that extend from the cellular membrane to the nucleus, governing oxidative stress, cell proliferation, apoptosis, differentiation, innate immunity, and inflammation (Guo et al., 2020). The MAPK pathway consists of MKK, MAPK kinase, and MAPK components. Recently, emerging members of the MAPK family, such as ERK1/2, ERK3/4, ERK5, ERK7/8, JNK 1/2/3, and p38 α/β/γ (ERK6)/δ), have been elucidated (Chen et al., 2019b). Within multicellular organisms, protein kinases participate in modulating cellular functions by adding phosphate groups to distinct serine and threonine positions on target protein substrates (Oh et al., 2019; Guo et al., 2020). Increasing evidence suggests that the activation of the MAPK cascade is intricately linked with the regulation of autophagy, which is associated with renal tubular injury and inflammation in the context of hyperuricemia (Bao et al., 2018; Wu et al., 2021). UA triggers apoptosis by disrupting the equilibrium between pro-apoptosis and anti-apoptosis proteins via the MAPK pathway. This leads to oxidative stress within kidney cells and contributes to inflammation (Jang et al., 2020). Consequently, there is an indication that genes responsible for mediating the positive regulation of the MAPK pathway might potentially contribute to hypouricemic effects (Jang et al., 2020).

Extracted from *Dioscorea polystachya* Turcz., the total saponin fraction exhibits the ability to ameliorate synovial tissue conditions. Additionally, it diminishes the protein expressions of phospho-MEK1/2, phospho-ERK1/2, phospho-JNK, and MKK4 in rats induced with MSU crystals. This process effectively mitigates inflammation in gout arthritis by inhibiting the MAPK signaling pathway (Zhou et al., 2020). Derived from the root bark of *Paeonia × suffruticosa* Andrews., paeonol (a phenolic compound), has been found to widespread use in TCM for various forms of arthritis (Liu et al., 2017a). Paeonol effectively hinders the expression of IL-1β induced by MSU and reduces the levels of phospho-MAPK (p-ERK, p-p38, and p-JNK), as well as phospho-IKKβ and phospho-IκBα. These findings suggest that paeonol curtails the activation of p-IKKβ through the MAPK pathway and prevents the degradation of p-IκBα. Consequently, paeonol functions as an anti-gout agent by regulating the MAPK signaling cascade (Chen et al., 2022). Astaxanthin, a xanthophyll carotenoid, exhibits the capability to lower the expression of phosphor-p38, phosphor-JNK, and phosphor-ERK1/2 within J774A.1 cells, synovial fibroblasts, and articular chondrocytes. As a consequence, this action inhibits the production of COX-2 proteins and NLRP3 inflammasomes, thereby enhancing its potential to mitigate MSU crystal-mediated arthritis through the deactivation of the MAPK pathway (Peng et al., 2020). *Phellodendron chinense* C.K.Schneid., a frequently employed herbal remedy for treating gout or hyperuricemia, effectively restores the over-expression of IL-6 and p-c-Jun proteins, along with mRNA levels of AKT, MAPK3, MAPK8, and TP53 within the renal tissues of hyperuricemia-afflicted mice. It further curbs UA production, exerting a nephroprotective effect through the inhibition of inflammatory and cell death, including MAPK (Xu et al., 2022a). Moreover, conventional Chinese herbal substances like *Clerodendrum trichotomum* Thunb. and *Rhus chinensis* Mill. manifest notable anti-inflammatory properties in cases of gout through their modulation of the MAPK signaling pathway (Jang et al., 2020; Ma et al., 2023).

Shiwei-Ruxiang powder is a customary remedy frequently employed in addressing gout and has demonstrated efficacy in clinical trials for preventing hyperuricemia and gout (Yu et al., 2022). Notably, Shiwei-Ruxiang powder has been observed to ameliorate renal impairment by diminishing CRE and BUN levels, while also counteracting the concentrations of pro-inflammatory agents like TNF-α and IL-1β, along with the expression of phospho-p38 MAPK, within hyperuricemia mice. This process is inherently connected to the MAPK signaling cascade (Li et al., 2022b). Wuwei-Shexiang pills, formulated with *Acorus calamus* L., *Moschus berezovskii* Flerov, *Aconitum carmichaelii* Debeaux., *Aucklandia lappa* (Decne) Decne., and *Terminalia chebula* Retz., exhibits the ability to alleviate ankle joint swelling and diminish TNF-α and IL-1β concentrations within MSU-induced mice air pouch lavage fluid. This is accomplished by restraining the levels of p-p38/p38 through the modulation of the MAPK signaling cascade, thus effectively mitigating gout-related inflammation (Bai et al., 2023).

## 3 The uric acid transporter of TCM in the treatment of gout

It has been documented that around 90% of individuals with hyperuricemia experience underexcretion-type hyperuricemia (Ota-Kontani et al., 2020). Both the intestine and the kidney contribute significantly to the elimination process of UA, with approximately 70%–75% being expelled through the renal pathway, while 20%–25% is excreted through the gastrointestinal tract. Importantly, UA is characterized by its polarity and does not readily traverse membranes. Consequently, the excretion of UA hinges on transporter proteins situated within renal proximal convoluted tubules and the intestinal environment (Dalbeth et al., 2021).

### 3.1 Renal uric acid transporter

Urate transport across the apical surface of the kidney, initiating in the lumen and reaching the cell, is mediated by specific transporters such as glucose transporter 9 (GLUT9/SLC2A9), organic anion transporter 4 (OAT4/SLC22A11), OAT10/SLC22A13, and urate anion transporter 1 (URAT1/SLC22A12) (Lu et al., 2019). ATP Binding Cassette Transporter C4 (ABCC4), ATP binding cassette transporter G2 (ABCG2), phosphate cotransporter type 1 (NPT1/SLC17A1), OAT1/SLC22A6, OAT2/SLC22A7, and OAT3/SLC22A8 function as secretory transporters (Dong and Dong, 2023). URAT1, characterized by the presence of 12 transmembrane structural domains, displays exclusive expression on the luminal membrane within the proximal renal tubule (Tan et al., 2017) and it is recognized as the primary apical urate/anion exchanger in humans, facilitating urate reabsorption. The deletion of the URAT1 gene results in a notable decrease in the reabsorption and excretion of UA, consequently precipitating renal hyperuricemia and the onset of gout (Kuo et al., 2017). GLUT9, found in both the basal and apical membranes of the renal proximal tubule, also accounts for the reabsorption of UA (Auberson et al., 2018), playing a crucial role in the renal process of UA excretion (Lüscher et al., 2022). Moreover, knockout mice of SLC2A9 demonstrate impaired enterocyte urate transport kinetics coupled with elevated serum urate concentration (Novikov et al., 2019). Present within the basolateral membrane of cells, OAT1 and OAT3 aid in the transportation of urate into proximal tubule cells, thereby contributing to the process of UA elimination (García-Nieto et al., 2022). This significance is highlighted by the observation of elevated serum UA levels in mice with knockout of OAT1 and OAT3, emphasizing the role of organic anion transporters in hyperuricemia pathogenesis (Wu et al., 2017). ABCG2, arising from a gene located at the chromosomal region linked to gout susceptibility on chromosome 4q, is widely recognized for its role as a potent urate secretion transporter with high capacity (Woodward, 2015). Its presence is notable on the apical membrane of proximal tubules in the human kidney (Lin et al., 2019), and it is similarly found in both the intestine and liver (Eckenstaler and Benndorf, 2021). The expression profile of ABCG2 within the kidney exhibits partial similarity to that of URAT1, implying a functional interplay between these two transporters in the handling of renal UA (Hoque et al., 2020). Research findings indicate that mice lacking the ABCG2 gene experience increased serum levels of UA, linked to escalated renal UA excretion and reduced UA excretion within the intestines (Toyoda et al., 2019).

### 3.2 Intestinal uric acid transporter

An additional path for UA elimination occurs within the intestines. ABCG2 is a pivotal regulator of urate excretion primarily in the gastrointestinal tract (Bhatnagar et al., 2016), signifying its crucial involvement in this physiological process (Yin et al., 2022). Impaired ABCG2 function notably raises the susceptibility to hyperuricemia and potentially gout, particularly among the younger population (Ye et al., 2022). In a well-established hyperuricemia mouse model achieved through the application of the uricase inhibitor potassium oxonate, there is a consistent elevation in the serum UA concentration within ABCG2-knockout mice (Kuo et al., 2017). Furthermore, studies have indicated that heightened ABCG2 expression was noticed in the intestines of nephrectomized rats, accompanied by reduced UA excretion. Surprisingly, even with this decrease, serum UA concentration showed no elevation, suggesting the potential involvement of the intestinal tract as an “extra-renal” route for urate elimination. This highlights the potential of intestinal urate excretion as a novel approach for gout and hyperuricemia treatment, offering the advantage of avoiding potential side effects like exacerbating kidney injury and urinary calculus formation.

Currently, it has been widely proven that TCM can effectively reduce UA in clinical practice. The multi-target function of TCM simultaneously affects multiple UA transporters, achieving the reduction of UA and the protection of the kidneys. Various herbal extracts used in TCM and natural bioactive compounds have been demonstrated to be beneficial in treating gout (Table 4).

## 4 Discussions and perspective

This comprehensive analysis has substantiated that Chinese medicine monomers, individual herbal remedies from Chinese tradition, and formulations of TCM offer significant assistance in gout management by impacting the expression and function of urate transporters. These encompass GLUT9, URAT1, OATs, and ABCG2, as well as their associated regulatory cascades like NLRP3, NF-κB, JAK/STAT, PI3K/AKT, and MAPK pathways. However, there are still several limitations must be considered in the gout therapy using TCM.

As we reviewed, these botanical drugs have shown positive anti-gout effects. Yet it is worth noting that the therapeutic effect of inhibiting inflammatory signaling pathways and uric acid transporters on gout has only been experimentally validated in cells and animals. Due to the differences between species, the therapeutic effects of some plant drugs with anti-inflammatory and reducing uric acid effects at the cellular and animal levels may not be satisfactory in the human, so more in-depth research is needed.

Another point to consider is that more studies are needed to confirm whether TCM can be effective targets for gout treatment. In recent literature, a compelling association between UA transporters and signaling pathways has come to light. UA drives excretion through the activation of signaling cascades involving NLRP3, PI3K/Akt and MAPK, leading to the augmentation of PDZK1 and ABCG2 expression, thereby mitigating the effects of hyperuricemia (Chen et al., 2018). The PI3K/Akt pathway stands out as a significant signaling route linked to transporter expression, capable of enhancing the levels of GLUT9 and URAT1 (Zhang et al., 2023a; Yang et al., 2023). Moreover, the depletion of GLUT9 led to the suppression of JAK2/STAT3 signaling, resulting in decreased release of pro-inflammatory molecules (Nie et al., 2021). Taken together, the mutual modulation between UA transporters and this signaling pathway may accomplish the dual objectives of diminishing UA level and safeguarding renal health and offer innovative avenues for advancing targeted gout therapy using TCM.

Notably, studies on the regulatory mechanisms the therapeutic applications of small molecule metabolites in gout by regulating protein functions are particularly lacking, which may be a weak direction for understanding the role in the pathogenesis and treatment of gout. Utilizing a metabolomics approach, researchers have employed a strategy to unveil the underlying metabolic mechanisms and plausible targets associated with TCM. Likewise, the interplay between metabolites and receptors offers an avenue to investigate potential therapeutic targets and the regulatory function of TCM within signaling pathways, facilitating internal signaling communication for addressing gout. A prime illustration lies in the distinctive involvement of purine metabolism in both inflammatory responses and immune system modulation in the context of gout. Notably, purinergic receptors, including P2X1, P2X7, P2Y2, and P2Y14, are found expressed across a wide spectrum of immune cells (Wang and Chen, 2018). ATP stimulation triggers immune cell activation, serving as a secondary pathogenic indicator in gout (Li et al., 2021c). Typically, altered ATP levels can trigger the P2X7R-NLRP3 signaling cascade, leading to the secretion of IL-1β linked to gout attacks (Tao et al., 2017). In contrast, ATP has the potential to convert into adenosine, resulting in the shift from the pro-inflammatory reaction through the P2Y and P2X receptor signaling routes to a more prevalent anti-inflammatory impact facilitated by the P1Y receptor signaling cascade. Hence, utilizing metabolomics strategy, a comprehensive exploration into distinct governing mechanisms within purine metabolism and the incorporation of diverse factors influencing both upstream and downstream processes can precisely ascertain the contribution of purine metabolism to gout flares. This endeavor offers viable avenues for strategic clinical interventions.

At present, we are engaged in clinical experimental investigations focusing on the role of biomarkers influenced by protein targets in the initiation of gout. Simultaneously, we are exploring the potential of utilizing TCM-regulated proteins to modulate metabolic pathways for gout management in mouse models. Furthermore, by combining and synthesizing the results of multiple studies that investigate TCM’s inhibitory effects on signaling pathways like NLRP3, along with its positive effects on gout, we are optimistic about substantial progress in the field of anti-gout TCM. This advancement involves a direct emphasis on proteins and biomarkers in the approaching phase, potentially leading to a smooth transition into clinical application and improving the current scenario of gout detection, prediction, and therapy.

## Figures and Tables

**Table:** 

**TCM**	traditional Chinese medicine
**UA**	uric acid
**MSU**	monosodium urate
**ATP**	adenosine triphosphate
**ADP**	adenosine diphosphate
**AMP**	adenosine monophosphate
**IMP**	inosine monophosphate
**NLRP3**	nod-like receptor thermal protein domain associated protein 3
**NF-κB**	nuclear factor κ-light-chain-enhancer of activated B cells
**interleukin**	IL
**LPS**	lipopolysaccharide
**PAMPs**	pathogen-associated molecular patterns
**TNF-α**	tumor necrosis factor-α
**Bcl-2**	B-cell lymphoma-2
**BAX**	Bcl-2 assaciated X protein
**IKK**	IκB kinase
**PGE2**	Prostaglandin E2
**COX-2**	cyclooxygenase-2
**MCP-1**	nonocyte chemoattractant protein-1
**PI3K**	Phosphatidylinositol 3 kinase
**AKT**	protein kinase B
**PDK1**	Phosphoinositide-dependent kinase-1
**AIF**	apoptosis-inducing factor
**PARP**	poly ADP-ribose polymerase
**P**	phosphorylated
**JAK**	Janus Kinase
**STAT**	signal transducer and activator of transcription
**IFN**	interferon
**SOCS3**	cytokine signaling 3
**MAPKs**	Mitogen-activated protein kinases
**JNK**	Jun N-terminal kinase
**ERK1/2**	extracellular signal-regulated kinases
**MKK4/7**	Mitogen-activated protein kinase kinase4/7
**MEK1/2**	mitogen-activated extracellular signal-regulated kinase1/2
**URAT1**	urate anion transporter 1
**GLUT9**	glucose transporter 9
**OAT**	organic anion transporter
**ABCG2**	ATP-binding cassette transporter G2
**ABCC4**	ATP Binding Cassette Transporter C4
**NPT1**	phosphate cotransporter type 1
